# Supplementary material for: Direct Photochemical Synthesis of Substituted Benzo[b]fluorenes
Source: Org Lett. 2024 Nov 25;26(48):10364–8. doi: 10.1021/acs.orglett.4c03978 (PMC11629382; doi:10.1021/acs.orglett.4c03978)
Supplement: Supplementary file 2 — ol4c03978_si_002.zip [file ol4c03978_si_002.zip › FID for Publication/5e/20240610_RC-2-C-5-98B-P2_01/RC-2-C-5-98B-P2_PROTON_20240610_02.fid/20240611_RC-2-C-5-98B_01/plots/RC-2-C-5-98B_NOESY_20240611_01_plot01.pdf]

RC-2-C-5-98B

Location	**13**	Pulse sequence	**NOESY**	Temperature	**25**	Suboperator	**RuairiCrawford**
Date collected	**2024-06-11**	Solvent	**dmso**	Spectrometer	**localhost.localdomain-vnmrs500**	Operator	**MBaumann**

P2

SAMPLE

date	Jun 11 2024
solvent	dmso
sample	20240611_RC-2-C-5-98B_01

ACQUISITION

sw	6410.3
at	0.150
np	1924
fb	4000
ss	32
d1	1.000
nt	8

2D ACQUISITION

sw1	6410.3
ni	256

TRANSMITTER

tn	H1
sfrq	499.724
tof	160.5
tpwr	58
pw	6.300

NOESY

mixN	0.500

PRESATURATION

satmode	n
wet	n

DECOUPLER

dn	C13
dm	nnn

FLAGS

hs	nn
sspul	y
PFGflg	y
hsglvl	5736

SPECIAL

temp	25.0
gain	40
spin	0

F2 PROCESSING

gf	0.069
gfs	not used
fn	2048

F1 PROCESSING

gf1	0.029
gfs1	not used
proc1	lp
fn1	2048

DISPLAY

sp	-546.0
wp	6410.3
sp1	-546.0
wp1	6410.3
rfl	546.0
rfp	0
rfl1	546.0
rfp1	0

PLOT

wc	142.6
sc	0
wc2	127.1
sc2	12.7
vs	235
th	2
ai	ph

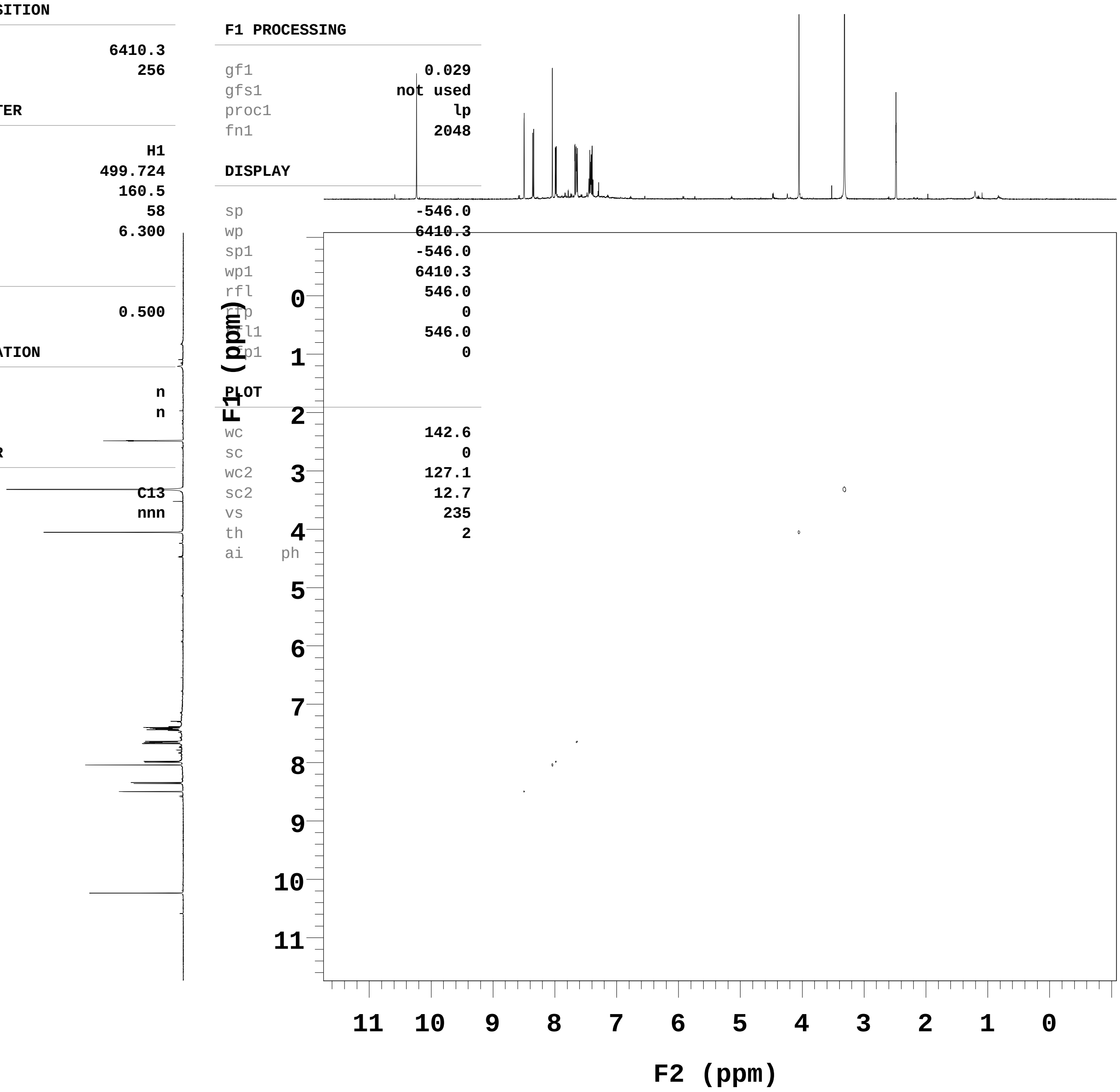

Plotname: RC-2-C-5-98B_NOESY_20240611_01_plot01

Data file /home/data/MBaumann/cscb500/RuairiCrawford/2024/20240611_RC-2-C-5-98B_01/RC-2-C-5-98B_NOESY_20240611_01.fid Plot date 2024-06-11
